# Wind Turbine Condition Monitoring Using the SSA-Optimized Self-Attention BiLSTM Network and Changepoint Detection Algorithm

**DOI:** 10.3390/s23135873

**Published:** 2023-06-25

**Authors:** Junshuai Yan, Yongqian Liu, Li Li, Xiaoying Ren

**Affiliations:** School of New Energy, North China Electric Power University, Beijing 102206, China

**Keywords:** wind turbine, condition monitoring, BiLSTM, self-attention, sparrow search algorithm, changepoint detection

## Abstract

Condition-monitoring and anomaly-detection methods used for the assessment of wind turbines are key to reducing operation and maintenance (O&M) cost and improving their reliability. In this study, based on the sparrow search algorithm (SSA), bidirectional long short-term memory networks with a self-attention mechanism (SABiLSTM), and a binary segmentation changepoint detection algorithm (BinSegCPD), a condition-monitoring method (SSA-SABiLSTM-BinSegCPD, SSD) used for wind turbines is proposed. Specifically, the self-attention mechanism, which can mine the nonlinear dynamic characteristics and spatial–temporal features inherent in the SCADA time series, was introduced into a two-layer BiLSTM network to establish a normal-behavior model for wind turbine key components. Then, as a result of the advantages of searching precision and convergence rate methods, the sparrow search algorithm was employed to optimize the constructed SABiLSTM model. Moreover, the BinSegCPD algorithm was applied to the predicted residual sequence to achieve the automatic identification of deterioration conditions for wind turbines. Case studies conducted on multiple wind turbines located in south China showed that the established SSA-SABiLSTM model was superior to other contrast models, achieving a better prediction precision in terms of RMSE, MAE, MAPE, and R^2^. The MAE, RMSE, and MAPE of SSA-SABiLSTM were 0.2543 °C, 0.3412 °C, and 0.0069, which were 47.23%, 42.19%, and 53.38% lower than those of SABiLSTM, respectively. The R^2^ of SABiLSTM was 0.9731, which was 4.6% higher than that of SABiLSTM. The proposed SSD method can detect deterioration conditions 47–120 h in advance and trigger fault alarm signals approximately 36 h ahead of the actual failure time.

## 1. Introduction

In recent years, wind energy, as a rapidly developing and inexhaustible clean renewable energy source, has become an indispensable force in addressing the world energy problems of fossil energy depletion and ecological environment deterioration [1,2,3]. By the end of 2022, according to the latest statistics [4] obtained from the Global Wind Energy Council (GWEC), the newly installed and cumulative capacities of wind turbines have achieved values up to 78 GW and 906 GW, respectively. However, most wind turbines are located in remote areas (e.g., mountains, deserts, and offshore) and are usually long-term, operating in severe weather conditions and complex geographical environments, which leads to frequent failures and even shutdowns [5]. Moreover, unexpected faults and shutdowns result in a higher operation and maintenance (O&M) cost and lower reliability of wind turbines, which may adversely influence the financial gains from wind farms and have a significant impact on the thriving wind power industry [6]. Statistically, the O&M cost of an onshore wind turbine accounts for approximately 10–15% of the total production cost, while this figure is as high as 20–30% for offshore wind turbines [7]. Therefore, to minimize the O&M cost and increase the safety and reliability of wind turbines, it is essential to investigate wind turbine-condition-monitoring (WTCM) technologies for the early detection of potential faults, thus avoiding secondary damages and even catastrophic accidents [8].

To date, extensive research on WTCM methods has been conducted by numerous scholars in the field, which can be generally divided into two categories: physical-model-based and data-driven-based approaches. Physical-model-based approaches, typically including constrained Kalman filter [9], parity equation [10], T–S fuzzy approach [11], and observed-based approach [12], have been widely applied in the WTCM field. Nevertheless, with the increasing scale and complexity of wind turbines, establishing precise mathematical models for their use becomes difficult, which severely restricts the use of physical-model-based methods in practical engineering tasks to a considerable extent.

In contrast, with the development of acquisition, transmission, and storage technologies, data-driven-based methods have become an attractive choice in the WTCM field, which only expands on the measured data instead of accurate physical or mathematical knowledge. Recently, numerous data-driven-based methods have been proposed in the literature and widely employed for WTCM methods, including vibration signal analysis [13,14,15,16], oil signal analysis [17], acoustic emission signal monitoring [18,19], electrical signal analysis [20], and others. However, the above-mentioned methods require the installation of additional signal acquisition equipment, which would result in a substantial improvement in the investment cost [21].

Practically, for most wind farms, the supervisory control and data acquisition (SCADA) system has been widely deployed to measure and record large high-dimensional operation data. These SCADA data contain continuous parameters (e.g., meteorological conditions, temperature, pressure, power, and electrical measurements) and discrete information (e.g., startups, shutdowns, fault records, etc.), which can reflect the wind turbine’s operation or health conditions in a timely manner. Meanwhile, due to the advantages of their powerful feature representation capacity, artificial intelligence (AI) algorithms (e.g., machine learning and deep learning) have achieved a widespread application and development in many fields, presenting technological advances in their storage and processing power. Consequently, based on AI algorithms, large numbers of WTCM methods using SCADA data have been proposed in the literature and proved to be effective and superior, and, at present, they are research hotspots in the field of wind power [22,23,24].

Recently, numerous SCADA-AI-based WTCM methods used for potential fault-detection purposes have been studied extensively in research, including support vector machines (SVMs), XGBoost, back-propagation neural networks (BPNNs), Gaussian process, restricted Boltzmann machine (RBM), auto-encoder (AE), and stacked denoising auto-encoder (SDAE). Dhiman H.S. et al. [25] proposed a data-driven anomaly detection approach for a wind turbine gearbox by using a twin support vector machine (TWSVM). Trizoglou P. et al. [26] utilized the extreme gradient-boosting (XGBoost) algorithm to construct a normal-behavior model of a wind turbine generator to detect deterioration. Sun P. et al. [27] established a generalized method for the detection of potential faults in wind turbines based on back-propagation neural networks (BPNNs) through the use of SCADA data. Infield D. et al. [28] constructed a SCADA-based potential fault-detection approach for wind turbines by using the Gaussian process (GP). Meyer A. et al. [29] explored a multi-target model as a novel method for detecting a wind turbine’s normal behavior. Yang W. et al. [30] designed an unsupervised early fault-detection approach for wind turbine anomaly detection purposes by applying a spatiotemporal pattern network (STPN) and stacked restricted Boltzmann machine (RBM). Renström N. et al. [31] designed a condition-monitoring framework based on an auto-encoder (AE) by using SCADA data and investigated the various hyperparameters that affect the model’s performance. By utilizing stacked denoising auto-encoders (SDAEs), Chen J. et al. [32] introduced a multivariate analysis method to detect early faults in wind turbines.

It has been proved in the research that the methods mentioned above are effective and have made great progress in the WTCM field. However, the methods mainly focus on the nonlinear correlation evident between different monitoring variables without considering the autocorrelation (i.e., temporal correlation) of every monitoring variable, which results in a limited model performance. In fact, SCADA data are essentially time series and monitor variable changes dynamically over time, therefore, they should be considered in the modeling process.

Recurrent neural networks (RNNs) are a class of neural networks that efficiently process sequential data with short-term memory capability. However, when the input sequence is relatively long, vanishing or exploding gradients are evident, which are also known as the long-term dependencies problem. The long short-term memory (LSTM) and gated recurrent unit (GRU) networks, as commonly used RNN variants, solve the problems of gradient explosion and gradient vanishing in traditional RNNs by introducing the gating mechanism. RNNs have been widely applied in the field of wind power generation, such as power prediction, early anomaly detection, and potential fault diagnosis. Zhang J. et al. [33] applied the long short-term memory network (LSTM) to predict the power of wind turbines and employed the Gaussian mixture model (GMM) to explore the distribution characteristics of the predicted residual. Lei J. et al. [34] designed a novel condition-monitoring framework by using an end-to-end LSTM network to mine the temporal features inherent in a multi-variate SCADA time series. By combining the auto-encoder neural network and LSTM network, Chen H. et al. [35] proposed a novel early anomaly-detection approach for wind turbine key components. The convolutional neural network (CNN) and gated recurrent unit (GRU) network were combined by Kong Z. et al. [36] to learn the spatial–temporal features inherent in SCADA data. The attention mechanism (AM) [37] can optimize resource allocations and make RNN models focus on more critical and highly relevant input features, thereby further improving the prediction accuracy of RNN models. A novel anomaly detection approach was developed by Xiang L. et al. [38] by using CNN and attention-based LSTM networks to monitor the condition of wind turbines. Case studies have demonstrated that the designed method was more effective and reliable in detecting potential anomalies for wind turbines. However, in the parameter initialization of the model training stage for the above-mentioned condition-monitoring models, the initial weight and bias parameters were randomly selected, which increases their potential to easily become stuck in local optima. In addition, numerous studies propose that Swarm Intelligence Optimization Algorithms (SIOA) [39,40,41,42] provide an effective solution for the iterative optimization process of model training.

To sum up, scholars have conducted numerous studies on the condition-monitoring and potential anomaly-detection methods of wind turbines from the aspects of data source, features extraction, model construction, and attention mechanism introduction; however, the following limitations still need to be resolved in the research.

(1) The previous studies mainly focus on the nonlinear relationships between different input variables, lack consideration of the input variable autocorrelation feature, and cannot fully mine the spatial–temporal features inherent in large high-dimensional SCADA time series.
(2) During the model training process, the existing normal-behavior models for wind turbines mostly adopt random model initialization parameters, without experiencing intelligent optimizations, and easily fall into the local minimum values.
(3) To date, the fixed or adaptive thresholds utilized in the existing studies for predicted error time series may result in missed detections or false alarms due to the overly large or too-small thresholds. Therefore, the accuracy and reliability of the anomaly detection method can still be enhanced by combining it with other statistical analytic techniques.

Consequently, to address the above-mentioned limitations, based on the sparrow search algorithm (SSA) [41], self-attention (SA) mechanism [37], bidirectional long short-term memory network (BiLSTM), and binary segmentation changepoint detection algorithm (BinSegCPD) [43,44], for the key components (e.g., main bearings, gearbox, and generator) of wind turbines, a novel WTCM method (SSA-SABiLSTM-BinSegCPD, SSD) is presented in this study, which can conduct real-time condition monitoring to detect potential anomalies, realize predictive maintenance, and reduce the O&M costs, with the contributions being summarized below.

(1) A novel normal-behavior model (SABiLSTM) for wind turbine key components is constructed by combining the self-attention mechanism and the BiLSTM network. The self-attention mechanism can make the model focus on input variables that have greater impacts on the output variable. The SABiLSTM can effectively mine the spatial–temporal features hidden in the SCADA time series. Compared with four contrast models (e.g., XGBoost, BPNN, LSTM, and BiLSTM), the SABiLSTM model achieved a superior prediction performance with better evaluation metrics (the lowest MAE, RMSE, and MAPE and the highest R^2^).
(2) The sparrow search algorithm (SSA) is employed to intelligently optimize the constructed SABiLSTM model for optimal initialization weights or bias parameters, which can considerably enhance the model’s overall performance and convergence rate. The comparative results, with two other optimization algorithms (i.e., particle swarm and crisscross optimization algorithms, denoted as PSO and CSO, respectively) [39,40], demonstrate that the introduced SSA algorithm performs better than the other two algorithms in terms of MAE, RMSE, MAPE, and R^2^.
(3) A hybrid anomaly detection strategy, consisting of the binary segmentation changepoint detection algorithm (BinSegCPD) and threshold alarm, is designed to automatically identify deterioration conditions and detect the potential anomalies in a wind turbine in advance. Two actual fault case studies of main bearings illustrated that the designed hybrid strategy can detect deterioration conditions 47–120 h in advance and trigger the fault alarm signals approximately 36 h ahead of the actual failure time.

The remainder of this paper is structured as follows: the framework of the designed SSD condition-monitoring method for wind turbines is briefly introduced in Section 2; a detailed description of the designed SSA-SABiLSTM model structure, as well as the corresponding methodologies, is provided in Section 3; Section 4 presents a hybrid anomaly detection strategy that consists of a binary segmentation changepoint detection algorithm (BinSegCPD) and threshold alarm; the proposed SSD method is validated using SCADA datasets acquired from multiple wind turbines located in southern China in Section 5; finally, a brief conclusion is presented in Section 6.

## 2. Proposed SSD Method

### 2.1. Overview of the Proposed SSD Method

The main work to be done in this study was to build a wind turbine normal-behavior model, which was employed to conduct the real-time condition monitoring and identify the early anomalies by applying a designed hybrid anomaly detection strategy.

The novelties of this study are that an SSA-SABiLSTM normal-behavior model, which can effectively mine the dynamic temporal characteristics of SCADA data by introducing the self-attention mechanism and can achieve a well-trained model with smaller training loss by introducing the SSA algorithm, was conducted for key components of wind turbines. Additionally, a hybrid anomaly detection strategy consisting of the changepoint detection algorithm and alarm threshold was designed to decrease the ratio of missed detections or false alarms.

**Figure 1:**
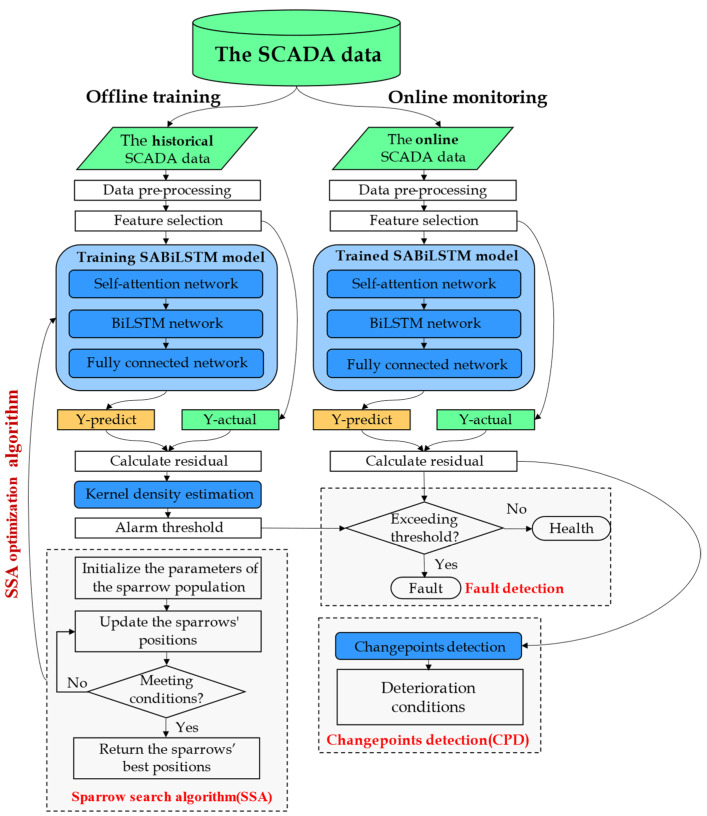
Framework of the proposed SSD condition-monitoring method.

As illustrated in Figure 1, the designed SSD wind turbine condition-monitoring (WTCM) method primarily comprises two stages: offline training and online monitoring. In order to capture the nonlinear and temporal dynamic properties of wind turbines under normal-state conditions, the key component of this framework was to build a normal-behavior model (NBM) of wind turbines based on the SABiLSTM network optimized by the sparrow search algorithm (SSA). A representative advantage of the designed method was that the modeling datasets only relied on historical health SCADA data, which could be easily deployed for the actual application where fault datasets were difficult or even impossible to acquire. Moreover, the primary principle behind this framework was that, according to the statistical analysis of the residuals between the measurements and predictions generated by the well-trained NBM, the residual change trend could indicate the possible deterioration conditions or potential faults. Generally, lower residuals would be produced by the well-trained NBM for normal SCADA operation data, whereas higher residuals were produced for abnormal or fault data. On the other hand, for anomaly detection strategies, in addition to the common threshold alarm, a binary segmentation changepoint detection (BinSeg) algorithm was further introduced to identify deterioration conditions of wind turbines, which could improve the timeliness and accuracy of the anomaly detection method. Therefore, the specific steps performed for each phase of the designed method can be summarized as follows.

Stage 1—Offline modeling. In this stage, based on the historical health SCADA data acquired by the SCADA system from multiple wind turbines that are operating in normal conditions, the proposed SSA-SABiLSTM normal-behavior model for key components of wind turbines were trained and tested. Specifically, firstly, the historical health-modeling dataset could be acquired after conducting data preprocessing (i.e., data cleaning and normalization) and variable selection processes on the raw SCADA datasets, which were collected from multiple wind turbines operating in normal conditions. The modeling dataset was then split into two sub-datasets: one for model training and the other for model testing. With the training sub-dataset, the designed SSA-SABiLSTM model could be trained to learn the nonlinear values between different variables and the temporal dynamic characteristics inherent in each variable when wind turbines operate normally. For the test sub-dataset, the prediction residuals could be computed by using the relevant measurements and the predictions generated by the well-trained SSA-SABiLSTM model. Furthermore, for normal SCADA data, by using the kernel density estimation algorithm, the probability density function (PDF) of prediction residuals could be calculated, so as to set the alarm threshold for potential early fault-detection purposes.

Stage 2—Online monitoring. In this stage, based on the well-trained SSA-SABiLSTM normal-behavior model, online SCADA data, and the hybrid strategies consisting of the changepoint detection algorithm and alarm threshold, the SSD method was used to conduct the real-time condition monitoring and early fault detection for wind turbine key components. Firstly, online monitoring SCADA data were preprocessed and fed into the well-trained SSA-SABiLSTM model to generate the predictions and corresponding predicted residuals. Then, the deterioration conditions of wind turbines could be automatically identified by using the BinSeg algorithm on the predicted residual sequence. Then, an early alarm could be triggered if the predicted residual value exceeded the alarm threshold value calculated in the offline training phase.

### 2.2. Data Preprocessing and Variable Selection Methods

#### 2.2.1. Data Preprocessing Method

It should be emphasized that the data preprocessing method (i.e., data cleaning and normalization) is an essential step in the WTCM method prior to constructing and training the normal-behavior model using SCADA data. Due to sensor errors or communication failures, a large number of invalid values or outliers may be generated and recorded by the SCADA system while a wind turbine continuously operates over a long period of time. Typically, these abnormal records severely influence the model’s overall performance; therefore, they should first be removed.

In this paper, the quartile algorithm (QA) was employed to perform data cleaning, and the interquartile range $I_{QR}$ can be calculated by Equation (1):

(1) $$I_{QR}=Q_{3}-Q_{1}$$

where $Q_{1}$ and $Q_{3}$ represent the first and third quartiles, respectively.

Then, the interquartile range $I_{QR}$ can be used to calculate the cleaning threshold of outliers by Equation (2):

(2) $$\left[T_{L},T_{U}\right]=\left[Q_{1}-{1.5I}_{QR},Q_{3}+{1.5I}_{QR}\right]$$

The data beyond the threshold $\left[T_{L},T_{U}\right]$ should be regarded as anomalies and eliminated from the raw SCADA dataset.

Furthermore, since different monitored variables generally present different value ranges, it is essential to reduce the original measurements to [0, 1], according to Equation (3), to eliminate the dimension affects and decrease the model training difficulty.

(3) $$X^{\prime }=\frac{X-X_{min}}{X_{max}-X_{min}}$$

where *X* is the data prior to normalization; $X_{max}$ is the maximum value of dataset *X*; and $X_{min}$ is the minimum value of dataset *X*.

#### 2.2.2. Variable Selection

Generally, the SCADA system records hundreds of monitored variable parameters. However, it is not necessary to use all variables for modeling purposes, because that improves the complexity of the wind turbine NBM and decreases its computing efficiency. Therefore, among all the monitored variables, those that present higher correlations with the target modeling variable (e.g., main bearing temperature) should be selected as the model input variables using some correlation calculation methods.

In this paper, the Pearson’s correlation coefficient was adopted to implement the variable selection process, which can be calculated by Equation (4):

(4) $$R_{p}=\frac{\sum_{i=1}^{N}\left(X_{i}-\overset{-}{X}\right)\left(Y_{i}-\overset{-}{Y}\right)}{\sqrt{\sum_{i=1}^{N}(X_{i}-\overset{-}{X}{)}^{2}}\sqrt{\sum_{i=1}^{N}(Y_{i}-\overset{-}{Y}{)}^{2}}}$$

where $\overset{-}{X}$ is the average value of *X* and $\overset{-}{Y}$ is the average value of Y.

## 3. Proposed SSA-SABiLSTM Model

### 3.1. Sparrow Search Algorithm

The sparrow search algorithm (SSA) proposed by Xue J. et al. [41] in 2020 is a swarm intelligence optimization approach, which is motivated by the group wisdom, foraging, and anti-predation behaviors of sparrows. Compared with other existing optimization algorithms, the SSA algorithm is superior in terms of its convergence speed, searching accuracy, and stability factors.

Specifically, from the perspective of biological characteristics, the SSA is designed based on the following assumptions. Sparrows generally can first be classified into two categories: producers and scroungers. Producers generally have a significant amount of energy reserve resources, which depend on the evaluation of the individual fitness values. They provide foraging directions and areas to scroungers through energetically searching for food sources. However, to improve their predation ratio, scroungers generally acquire food through following, monitoring, and competing with producers.

As for the mathematics, according to Equations (5) and (6), the positions and fitness values of sparrows can be denoted as matrix *X* and vector *F*(*X*), respectively.

(5) $$X=\left[\begin{array}{ccc} x_{1,1} & \cdots & x_{1,d}\\ \vdots & \ddots & \vdots \\ x_{n,1} & \cdots & x_{n,d} \end{array}\right]$$

(6) $$F(X)=\left[\begin{array}{ccc} {f(x}_{1,1} & \cdots & x_{1,d})\\ \vdots & \ddots & \vdots \\ f(x_{n,1} & \cdots & x_{n,d}) \end{array}\right]$$

where n represents the number of sparrows and d stands for the dimension of the optimized variables.

As mentioned above, producers generally possess higher fitness values and have the responsibility of searching for food and guiding the movement direction of the sparrow population. Therefore, compared with the scroungers, producers have access to a wider range of locations to search for food, and the positions of the producers can be updated by Equation (7):

(7) $$X_{i,j}^{t+1}=\left\{\begin{array}{cc} X_{i,j}^{t}\cdot exp\left(\frac{-i}{\alpha \cdot iter_{max}}\right) & ,R_{2}<ST\\ X_{i,j}^{t}+Q\cdot L & ,R_{2}\geq ST \end{array}\right.$$

where $iter_{max}$ indicates the maximum iteration number; α∈(0,1] and *Q*~*N* (0, 1) are random numbers; *L* indicates a 1 × *d* matrix whose elements are all 1; $R_{2}\in (0,1]$ represents the alarm threshold; and ST∈(0.5,1] represents the safety threshold. 

The positions of the scroungers can be updated according to Equation (8):

(8) $$X_{i,j}^{t+1}=\left\{\begin{array}{cc} Q\cdot exp\left(\frac{{X^{t}}_{worst}-X_{i,j}^{t}}{i^{2}}\right) & ,i>n/2\\ X_{P}^{t+1}+\left|X_{i,j}^{t}-X_{P}^{t+1}\right|\cdot A^{+}\cdot L & ,i\leq n/2\\ A^{+}=A^{T}(AA^{T}{)}^{-1} \end{array}\right.$$

where $X_{P}^{t}$ represents the best positions occupied by the producers; ${X^{t}}_{worst}$ represents the global worst positions at iteration *t*; and *A* indicates a 1 × *d* matrix whose elements are randomly assigned as 1 or −1. 

We assume that some sparrows, accounting for approximately 10% to 20% of the total population, have the ability to notice danger. Additionally, these sparrows’ beginning placements within the population are selected at random, which can be expressed as Equation (9):

(9) $$X_{i,j}^{t+1}=\left\{\begin{array}{cc} {X^{t}}_{best}+\beta \left|X_{i,j}^{t}-{X^{t}}_{best}\right| & ,f_{i}>f_{g}\\ X_{i,j}^{t}+K\left(\frac{\left|X_{i,j}^{t}-{X^{t}}_{worst}\right|}{\left(f_{i}-f_{w}\right)+\varepsilon }\right) & ,f_{i}=f_{g} \end{array}\right.$$

where ${X^{t}}_{best}$ represents the global optimal positions at iteration t; K∈[−1,1] and *β*~*N* (0, 1) are random numbers; ε represents an extremely small constant for avoiding a zero-division error; $f_{i}$ indicates the fitness value of the ith sparrow; $f_{g}$ indicates the current global best fitness value; and $f_{w}$ indicates the current worst fitness value.

As described above, the specific procedures of the sparrow search algorithm can be summarized as the pseudo-code displayed in Algorithm 1.
**Algorithm 1.** Sparrow search algorithm (SSA)**Input:** *I*:  the maximum iterations *N_P_*:  the number of producers *N_D_*: the number of sparrows who perceive danger $R_{2}$:  the alarm value *n*:  the number of sparrows Initialize a population of *n* sparrows and define its relevant parameters. **Output**: $X_{best}$, $f_{g}$. 1: **while** (*t* < *I*) 2:  search for the best and worst individuals of the sparrow population by ranking the fitness values. 3:  $R_{2}$ = *rand*(1) 4:  **for**
*i* = 1: *N_P_*
**do** 5:   Updating the sparrow’s position by Equation (3); 6:  **end for**
7:  **for** i = (*N_P_* + 1): n **do**
8:   Updating the sparrow’s position by Equation (4); 9:  **end for**
10:  **for** *l* = 1: *N_D_*
**do**
11:   Updating the sparrow’s position by Equation (5); 12:  **end for**
13:  Obtain the current new position; 14:  If the new position is better than before, update it; 15:  *t* = *t* + 1 16: **end while**
17: **return** $X_{best}$, $f_{g}$


### 3.2. Structure and Theory of the Constructed SABiLSTM Model

**Figure 2:**
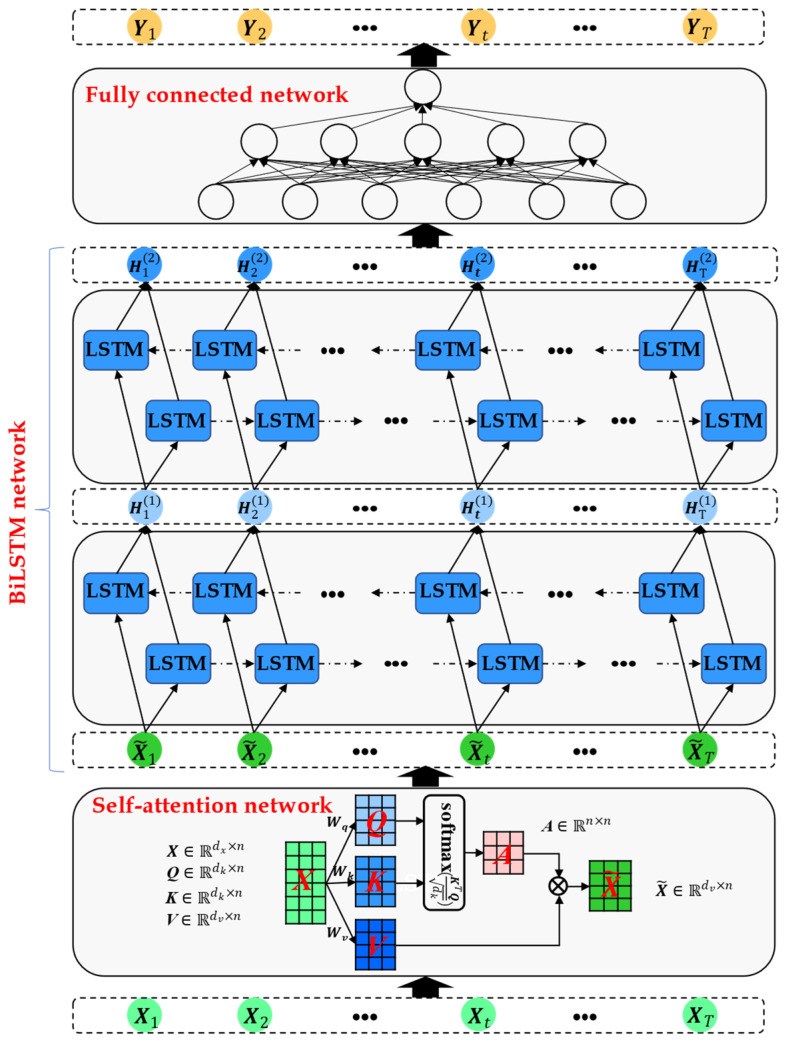
Structure of the proposed SABiLSTM model.

The structure of the constructed SABiLSTM model displayed in Figure 2 mainly contains three parts: the self-attention, BiLSTM, and fully connected networks. Specifically, for minibatches of historical health or online datasets (i.e., $X_{1},X_{2},\ldots ,X_{T}$) acquired after the data preprocessing stage, the weighted time-series (i.e., ${\tilde{X}}_{1},{\tilde{X}}_{2},\ldots ,{\tilde{X}}_{T}$) values can be calculated through the self-attention network, and the detailed calculated process of the self-attention mechanism can be observed in Section 3.2. Then, the weighted time-series (i.e., ${\tilde{X}}_{1},{\tilde{X}}_{2},\ldots ,{\tilde{X}}_{T}$) values, as model inputs, are fed into the BiLSTM network to generate the hidden variables time-series (i.e., $H_{1}^{\left(2\right)},H_{2}^{\left(2\right)},\ldots ,H_{T}^{\left(2\right)}$) values, which can be used as the inputs for the fully connected network to obtain the final target outputs (e.g., main bearing temperature). The detailed theory of the BiLSTM network is described in Section 3.2.3.

#### 3.2.1. Self-Attention Mechanism

Self-attention (SA), also known as intra-attention [37], is an attention mechanism linking different positions of a time series so as to calculate the attention weights of the time series. Recently, the self-attention mechanism has been widely applied in different tasks, including abstractive summarization, reading comprehension, and textual entailment. 

**Figure 3:**
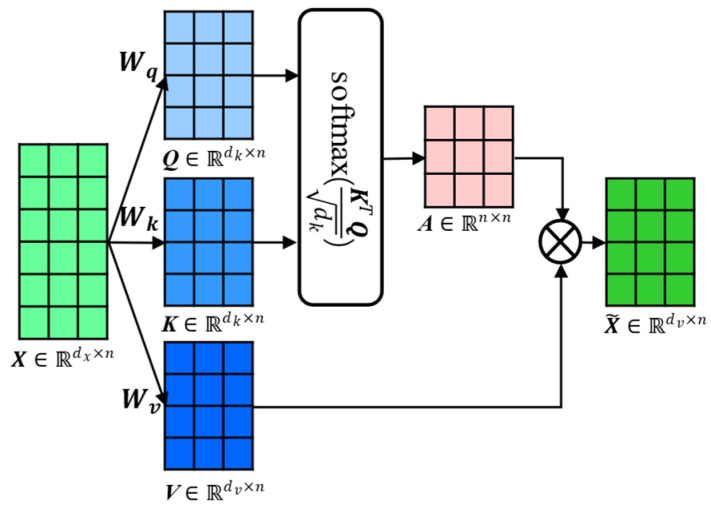
Calculation process of the self-attention mechanism.

Figure 3 presents the calculation process of the self-attention mechanism. Specifically, the self-attention function can be described as mapping a query and a set of key-value pairs to an output, where the query, keys, values, and output are all vectors. The output is computed as a weighted sum of the values, where the weight assigned to each value is computed by a compatibility function of the query with the corresponding key. In practice, we compute the attention function on a set of queries simultaneously, packed together into a matrix Q. The keys and values are also packed together into matrices K and V, as can be observed in Figure 3. The input matrix X was first transformed into matrices Q, K, and V, then a SoftMax function was applied on matrices Q and K to compute the attention weights matrix A, which was used to compute the output by multiplying with matrix V.

Mathematically, assume that the inputs sequence is denoted as $X{=[x}_{1}{,\ldots ,x}_{N}]\in R^{d_{x}\times n}$ and the outputs sequence is denoted as $\tilde{X}{=[\tilde{x}}_{1}{,\ldots ,\tilde{x}}_{N}]\in R^{d_{v}\times n}$. Then, the input $x_{i}\in R^{d_{x}}$, query $q_{i}\in R^{d_{k}}$, key $k_{i}\in R^{d_{k}}$, and value $v_{i}\in R^{d_{v}}$ vectors can be acquired through the linear mapping process. Moreover, for the whole input sequence X, three mapping matrices (i.e., *Q*, *K*, and *V*) and the output matrix $\tilde{X}$ can be calculated according to Equations (10)–(13). In this study, we selected a scaled dot-product as the attention scoring function.

(10) $$Q=W_{q}X\in R^{d_{k}\times n}$$

(11) $$K=W_{k}X\in R^{d_{k}\times n}$$

(12) $$V=W_{v}X\in R^{d_{v}\times n}$$

(13) $$\tilde{X}=VA=Vsoftmax\left(\frac{K^{T}Q}{\sqrt{d_{k}}}\right)\in R^{d_{v}\times n}$$

where $W_{q}\in R^{d_{k}\times d_{x}}$, $W_{k}\in R^{d_{k}\times d_{x}}$, and $W_{v}\in R^{d_{v}\times d_{x}}$ are parameter matrices; $Q{=[q}_{1}{,\ldots ,q}_{N}]$ is a query vector matrix; $K{=[k}_{1}{,\ldots ,k}_{N}]$ is a key vector matrix; $V{=[v}_{1}{,\ldots ,v}_{N}]$ is a value vector matrix; A is the attention matrix; and SoftMax is the normalization function.

#### 3.2.2. Long Short-Term Memory Network

Recurrent neural networks (RNNs, including RNN, BRNN, LSTM, and GRU) are a family of artificial neural networks that are good at processing sequential or time-series data due to their short-term memory capability. Among the many RNN variants available in the research, by introducing gate mechanisms, long short-term memory (LSTM) networks can effectively address the problem of gradient disappearance or gradient explosion existing in deep recurrent neural networks. 

**Figure 4:**
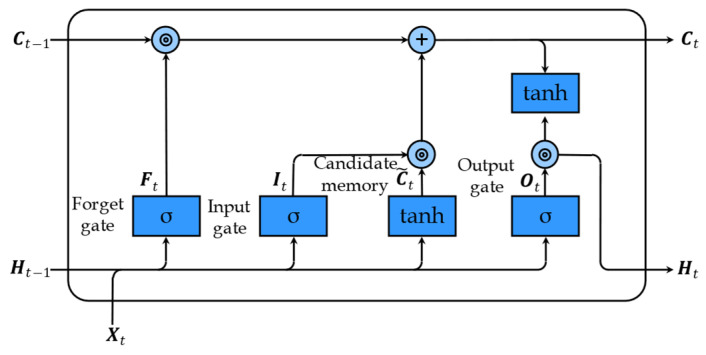
Intra-calculation process of an LSTM recurrent unit.

Figure 4 displays the intra-calculation process of an LSTM recurrent unit. As can be observed in Figure 4, compared with conventional RNN, LSTM introduces three gates, i.e., forget gate $F_{t}$, input gate $I_{t}$, and output gate $O_{t}$. The forget gate determines the information ratio that the memory cell needs to discard, the input gate determines the information ratio that the candidate memory cell needs to reserve, and the output gate determines the information ratio that the memory cell needs to pass to the hidden state.

Mathematically, we assume that the input matrix is a minibatch $X_{t}\in R^{n\times d}$ at a specific time-step t, and the memory cell and hidden state of the previous time-step are $C_{t-1}\in R^{n\times h}$ and $H_{t-1}\in R^{n\times h}$, respectively. Then, the forget gate $F_{t}$, input gate $I_{t}$, output gate $O_{t}$, candidate memory cell ${\tilde{C}}_{t}$, memory cell $C_{t}$, and hidden state $H_{t}$ can be computed according to Equations (14)–(19):

(14) $$F_{t}=\sigma \left(X_{t}W_{xf}+H_{t-1}W_{hf}+b_{f}\right)$$

(15) $$I_{t}=\sigma \left(X_{t}W_{xi}+H_{t-1}W_{hi}+b_{i}\right)$$

(16) $$O_{t}=\sigma \left(X_{t}W_{xo}+H_{t-1}W_{ho}+b_{o}\right)$$

(17) $${\tilde{C}}_{t}=\tanh\left(X_{t}W_{xc}+H_{t-1}W_{hc}+b_{c}\right)$$

(18) $$C_{t}=F_{t}⨀C_{t-1}+I_{t}⨀{\tilde{C}}_{t}$$

(19) $$H_{t}=O_{t}⨀\tanh\left(C_{t}\right)$$

Where $W_{xf}$, $W_{xi}$, $W_{xo}$, $W_{xc}\in R^{d\times h}$, $W_{hf}$, $W_{hi}$, $W_{ho}$, and $W_{hc}\in R^{h\times h}$ are weight parameter matrices; $b_{f}$, $b_{i}$, $b_{o}$, and $b_{h}\in R^{1\times h}$ are bias parameter matrices; n and d indicate the number of examples and the number of inputs in each example, respectively; h indicates the number of hidden units; σ represents the sigmoid function; and the symbol ⨀ represents the Hadamard product operator.

#### 3.2.3. Bidirectional Long Short-Term Memory Networks

**Figure 5:**
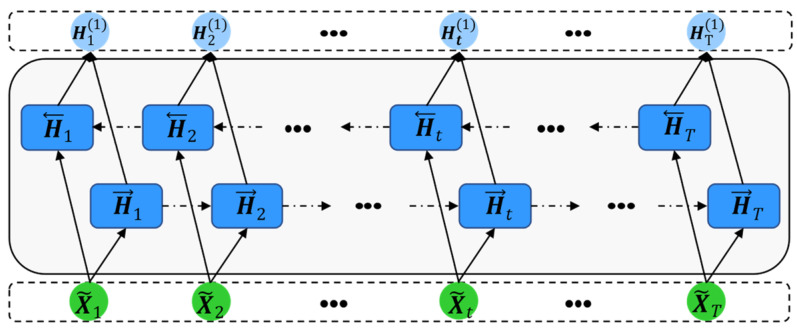
Structure of the BiLSTM network.

A bidirectional long short-term memory network (BiLSTM) is a neural network that consists of two LSTM networks with identical structures but opposite propagation directions. Figure 5 illustrates the typical structure of a one-hidden-layer BiLSTM network, with ${\vec{H}}_{t}$ standing for the state of the forward-LSTM network that moves forward through time and ${\overset{\leftarrow }{H}}_{t}$ standing for the state of the backward-LSTM network that moves backward through time. The special structure of BiLSTM can make the output $H_{t}^{\left(1\right)}$ learn a feature representation that depends on both the past and the future.

Mathematically, for a given minibatch input $X_{t}\in R^{n\times d}$, the forward ${\vec{H}}_{t}$ and backward ${\overset{\leftarrow }{H}}_{t}$ hidden states can be calculated by Equations (20) and (21):

(20) $${\vec{H}}_{t}=\emptyset \left(X_{t}W_{xh}^{\left(f\right)}+{\vec{H}}_{t-1}W_{hh}^{\left(f\right)}+b_{h}^{\left(f\right)}\right)$$

(21) $${\overset{\leftarrow }{H}}_{t}=\emptyset \left(X_{t}W_{xh}^{\left(b\right)}+{\overset{\leftarrow }{H}}_{t+1}W_{hh}^{\left(b\right)}+b_{h}^{\left(b\right)}\right)$$

where $W_{xh}^{\left(f\right)}$, $W_{hh}^{\left(f\right)}$, $W_{xh}^{\left(b\right)}$, and $W_{hh}^{\left(b\right)}$ are weight parameter matrices; $b_{h}^{\left(f\right)}$ and $b_{h}^{\left(b\right)}$ are bias parameter matrices; and ∅ represents the hidden-layer activation function.

Then, the hidden state value $H_{t}$ can be obtained by concatenating the forward and backward hidden states (i.e., ${\vec{H}}_{t}$ and ${\overset{\leftarrow }{H}}_{t}$) and provided to the output layer to compute the output $H_{t}^{\left(1\right)}$ (q indicates number of outputs):

(22) $$H_{t}=\left[{\vec{H}}_{t},{\overset{\leftarrow }{H}}_{t}\right]$$

(23) $$H_{t}^{\left(1\right)}=H_{t}W_{hq}+b_{q}$$

where $W_{hq}$ is the weight parameter matrix and $b_{q}$ is the bias parameter vector.

Additionally, in the deep BiLSTM networks with multiple hidden layers, the intermediate hidden state $H_{t}^{\left(1\right)}$ is supplied as the input value to the subsequent bidirectional layer.

#### 3.2.4. Evaluation Metrics

Four commonly used evaluation metrics—root mean square error (RMSE), mean absolute error (MAE), mean absolute percentage error (MAPE), and determination coefficient (R^2^)—were used to assess the effectiveness and superiority of the normal-behavior models for wind turbines, which can be calculated according to Equations (24)–(27):

(24) $$MAE=\frac{1}{N}\sum_{t=1}^{N}\left|x_{t}-x_{t}^{\prime }\right|$$

(25) $$RMSE=\sqrt{\frac{1}{N}\sum_{t=1}^{N}{\left(x_{t}-x_{t}^{\prime }\right)}^{2}}$$

(26) $$MAPE=\frac{1}{N}\sum_{t=1}^{N}\left|\frac{x_{t}-x_{t}^{\prime }}{x_{t}}\right|$$

(27) $$R^{2}=1-\frac{\sum_{t=1}^{N}{\left(x_{t}-x_{t}^{\prime }\right)}^{2}}{\sum_{t=1}^{N}{\left(x_{t}-\overset{-}{x}\right)}^{2}}$$

where $x_{t}$ represents the measurement; $x_{t}^{\prime }$ represents the prediction; and $\overset{-}{x}$ represents the mean of the measurements.

## 4. Wind Turbine Condition Monitoring

Based on the historical health SCADA data and designed SSA-SABiLSTM network, the normal-behavior model for key components or subsystems can be constructed offline to learn the dynamic spatial–temporal characteristics of wind turbines under normal conditions. Then, the well-trained SSA-SABiLSTM model can be used to implement real-time wind turbine condition-monitoring (WTCM) activity through the use of online SCADA data. Typically, for SCADA datasets collected from wind turbines operating under normal conditions, the residuals generated by the SSA-SABiLSTM model are small with stable fluctuations, while the residuals are large for abnormal conditions. Therefore, through the real-time statistical analysis applied to the predicted residual, the deterioration conditions could be automatically identified, and the potential faults could be captured in advance.

In this section, a hybrid anomaly detection strategy, consisting of the binary segmentation changepoint detection algorithm (BinSegCPD) and threshold alarm, is introduced to automatically identify deterioration conditions and detect early potential faults of wind turbines in advance.

### 4.1. Binary Segmentation Changepoint Detection Algorithm

Changepoint detection [45], first proposed in 1954 [46], is the process of identifying changes in univariate or multivariate time series. In addition to generating significant activity in the fields of statistics and signal processing, it has also had a significant impact on several application areas, including voice processing, financial analysis, bioinformatics, climatology, network traffic data analysis, and complex system monitoring.

Specifically, for a given time sequence $y={\left\{y_{t}\right\}}_{t=1}^{T}$ that is divided into K + 1 sub-sequences by changepoints set $J={\left\{t_{1},t_{2},\cdots ,t_{K}\right\}}_{K\leq T}$, the goal of a changepoint detection algorithm is to determine the optimal changepoints set $\hat{J}$ through minimizing the quantitative criterion $V\left(J,\;y\right)$ according to Equation (28):

(28) $$\underset{J}{min}V\left(J,\;y\right)=\underset{J}{min}\sum_{d=1}^{D}\sum_{k=1}^{K}C\left(y_{t_{k}:t_{k+1}}^{d}\right)+pen\left(J\right)$$

where D denotes the dimension of the time series; K denotes the number of changepoints; T denotes the length of the time series; $y_{t_{k}:t_{k+1}}$ refers to a sub-sequence of the time sequence; C (·) stands for the cost function; and $pen\left(J\right)$ stands for a constraint penalty term, which should be added to balance the number of obtained changepoints for an undetermined K.

Consequently, a changepoint detection algorithm mainly contains three components: a search method for finding J**,** a cost function C (·), and a penalty term $pen\left(J\right)$ when K is undetermined.

In terms of the search method, the binary segmentation algorithm [43], denoted as BinSeg, was adopted to obtain the optimal changepoints set $\hat{J}$ in this study.

Based on BinSeg, the first changepoint estimate ${\hat{t}}^{\left(1\right)}$ of time sequence y can be calculated by Equation (29):

(29) $${\hat{t}}^{\left(1\right)}=\underset{1\leq t<T-1}{argmin}V\left(T=\left\{t\right\}\right)=\underset{1\leq t<T-1}{argmin}C\left(y_{0:t}\right)+C\left(y_{t:T}\right)$$

Sequence y is then divided in half at position ${\hat{t}}^{\left(1\right)}$, and the same process is performed on the resulting sub-sequences until a stopping requirement is satisfied.

As for the cost function, the least squares deviation was employed in this paper, which measures the mean shifts in a time sequence, as presented in Equation (30):

(30) $$C\left(y_{I}\right)=\sum_{i\in I}{\left\|y_{i}-\overset{-}{y}\right\|}^{2}$$

where $y_{I}$ represents the sub-sequence set and $\overset{-}{y}$ stands for the mean value of sub-sequence $y_{i}$.

### 4.2. Alarm Threshold

In this study, the probability density function (PDF) of the prediction residual for wind turbines operating in normal conditions was calculated using the kernel density estimation (KDE) method, as presented in Equation (31):

(31) $$f\left(r\right)=\frac{1}{Nh}\sum_{i=1}^{N}K(\frac{r-r_{i}}{h})$$

where h denotes the smoothing parameter; N indicates the total number of samples; and K(·) stands for the kernel function, which is subject to $K\left(x\right)\geq 0$ and $\int_{-\infty }^{+\infty }K\left(x\right)dx=1$.

Then, for a given confidence α, the alarm threshold of the wind turbine condition-monitoring (WTCM) method employed for potential fault-detection purposes can be determined by Equation (32):

(32) $$\alpha =P\left(r<r*\right)=\int_{0}^{r*}f\left(r\right)dr$$

## 5. Case Study

### 5.1. Dataset Description

The datasets studied in this paper were gathered from eight wind turbines (EN-70/1.5) in a wind farm located in south China, which contained thirty-three 1.5 MW wind turbines. The type of main bearing used is spherical roller bearing and their manufacturer/model is SKF 240/600CA. The detailed datasets’ descriptions are displayed in Table 1.

As can be observed in Table 1, health training dataset A, which contains 153,162 data records collected from 3 wind turbines (No.11, No.15, and No.19) during the period of 1/1/2020–1/1/2021, was employed to create the normal-behavior model (NBM) for wind turbine main bearings. Health test dataset B, including 52,215 data records collected from 3 wind turbines (No.20, No.23, and No.26) during the periods of 1/1/2020–4/20/2020, 5/1/2020–9/20/2020, and 8/1/2020–11/18/2020, was employed to validate the performance of the well-trained main bearing NBM. Fault dataset C, consisting of 9097 data records collected from 2 failure wind turbines (i.e., No.14 and No.27) during the periods of 5/9/2020–6/9/2020 and 7/17/2020–8/17/2021, was adopted to verify the effectiveness of the designed SSD condition-monitoring approach for potential fault-detection purposes.

**Figure 6:**
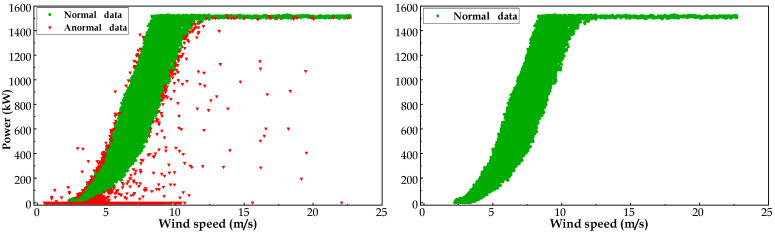
Result of data cleaning for WT No.11.

It should be noted that, prior to being used to establish the main bearing NBM, the training dataset A and test dataset B needed to experience data cleaning in order to obtain the health datasets. According to the data preprocessing algorithm presented in Section 2.2.1, the result of the data cleaning stage for WT No.11 is presented in Figure 6. As can be observed in Figure 6, through the quartile algorithm (QA), abnormal SCADA data (e.g., shutdowns, sensor errors, and communication failures) displayed as red scatter were removed to acquire the historical health data.

When taking the model performance and computational efficiency into account, it is essential to conduct variable selection using the Pearson’s correlation coefficient described in Section 2.2.2 by selecting the variables that have higher correlation coefficients with main bearing temperature values. The result of the variable selection can be observed in Table 2, and 16 variables were reserved as model inputs, whereas the main bearing temperature was the model output.

### 5.2. Model Validation

#### 5.2.1. The SABiLSTM Model

In this section, based on health training dataset A and health test dataset B, the proposed SABiLSTM main bearings NBM was trained and tested. Four commonly used contrast models for time series, i.e., XGBoost, BPNN, LSTM, and BiLSTM, were established for comparison. Meanwhile, the learning rate and estimator number of XGBoost were set as 0.1 and 100, respectively. The structure of the BPNN was designed as 16-32-16-8-1. As can be observed in Table 3, the hyper-parameters of the remaining RNN (i.e., LSTM, BiLSTM, and SABiLSTM) models were set to the identical values for comparison.

For health test dataset B, the quantitative evaluation metrics of the predicted results of well-trained main bearing NBMs mentioned above are presented in Table 4 and Table 5.

As shown in Table 4 and Table 5, when compared to XGBoost and BPNN, the RNN models presented superior performances with lower MAE, RMSE, and MAPE values and a higher R^2^ value. The reason for this was that the RNN models were better at processing time-series information and could extract the temporal features inherent to the wind turbine operating SCADA data. Due to the added backward layer, the BiLSTM model outperformed the LSTM network in terms of four mean metrics, which are presented in Table 5. The MAE, RMSE, and MAPE values of the BiLSTM were 9.68%, 9.19%, and 4.65% lower than those of LSTM, respectively, while the R^2^ value was 2.45% higher than that of the LSTM network. Meanwhile, it can be easily observed in Table 4 and Table 5 that, with the introduction of the self-attention mechanism, the prediction performance of the BiLSTM network improved to a significant extent. The MAE, RMSE, and MAPE values of the SABiLSTM network were 0.4819 °C, 0.5902 °C, and 0.0148, which were 4.82%, 11.02%, and 1.77% lower than those of the BiLSTM network, respectively. The R^2^ value of SABiLSTM was 0.9271, which was 2.91% higher than that of the BiLSTM network.

**Figure 7:**
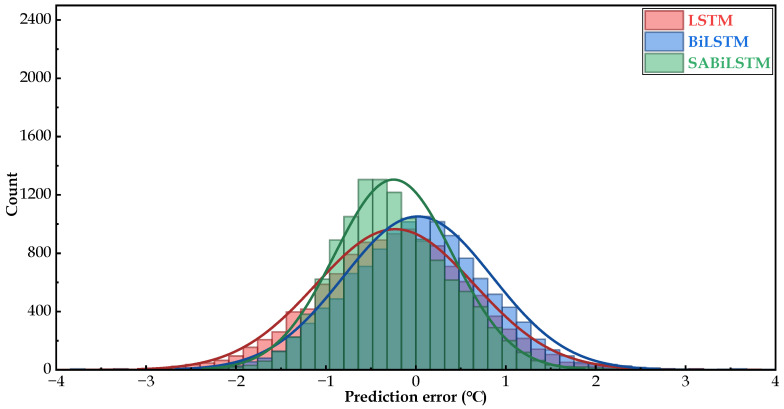
Probability density distribution of prediction errors of different NBMs for the main bearing temperature of WT No.20.

The probability density distribution of the predicted residual of the RNN models (i.e., LSTM, BiLSTM, and SABiLSTM) for WT No.20 is presented in Figure 7. As displayed in Figure 7, all PDF curves were sharp around the zero-error interval, indicating a high proportion of small prediction residuals. The PDF curved shape of the SABiLSTM network was better than LSTM and BiLSTM networks, which was consistent with the quantitative evaluation results in terms of MAE, RMSE, and MAPE values, as shown in Table 4 and Table 5.

**Figure 8:**
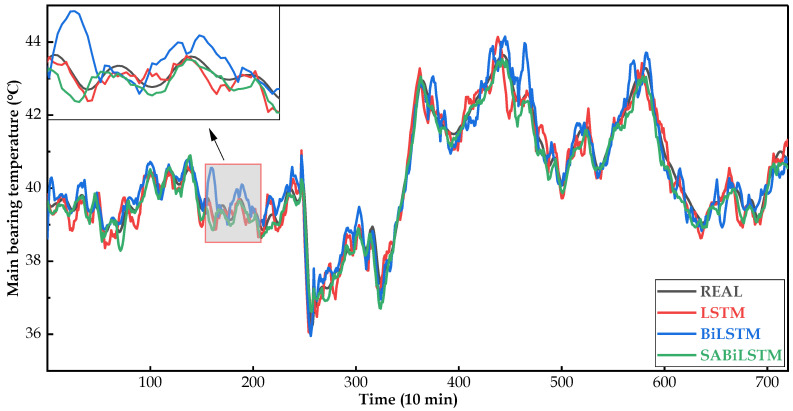
Main bearing predicted temperature values of different NBMs for WT No.20.

**Figure 9:**
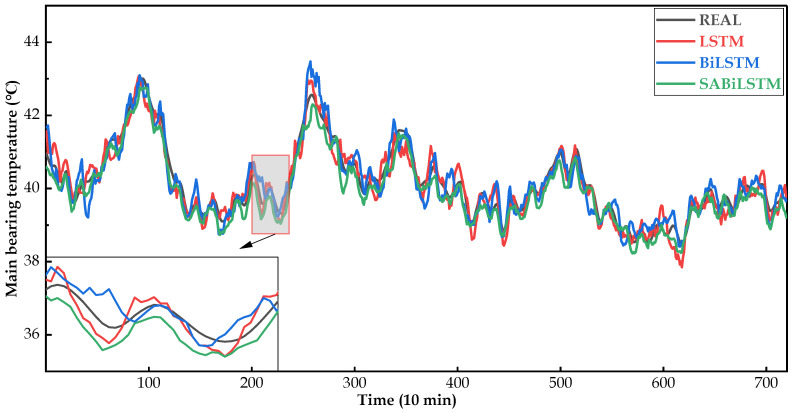
Main bearing predicted temperature values of different NBMs for WT No.23.

**Figure 10:**
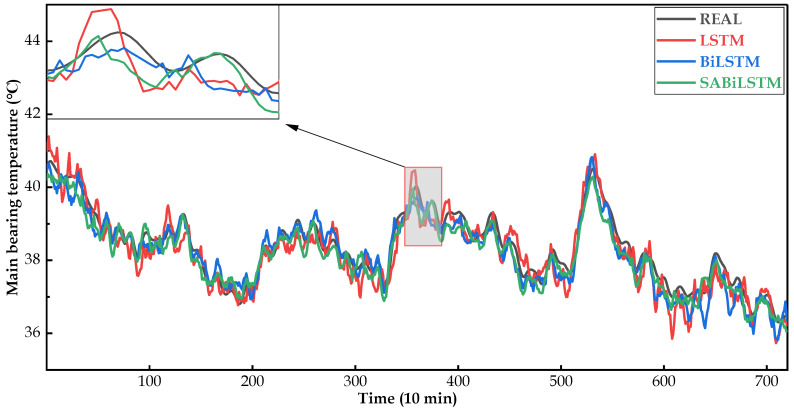
Main bearing predicted temperature values of different NBMs for WT No.26.

Figure 8, Figure 9 and Figure 10 intuitively display the main bearing predicted temperature values of the RNN models (i.e., LSTM, BiLSTM, and SABiLSTM) for WTs No.20, No.23, and No.26.

It can be easily observed in Figure 8, Figure 9 and Figure 10, compared with the LSTM and BiLSTM networks, the predicted values of the main bearing temperature using the proposed SABiLSTM model were closer to the real values of WTs No.20, No.23, and No.26, which validated the effectiveness and superiority of the SABiLSTM network and corresponded to the quantitative evaluation result of R^2^ listed in Table 4 and Table 5.

#### 5.2.2. The SSA-SABiLSTM Model

As described in Section 3.1, the model performance and convergence speed values of the NBMs for wind turbine main bearings could be significantly improved by using intelligent optimization algorithms (IOAs) to optimize the initialization weights or bias parameters. Therefore, in this paper, based on the proposed SABiLSTM network presented in Section 3.2, the optimal initialization weights and bias parameters could be acquired by introducing the sparrow search algorithm (SSA), one of the IOAs, to increase the training speed and prediction precision results.

To validate the feasibility of the early optimization of the model initialization parameters (weights and bias) using IOAs, and to compare the optimization effects of different IOAs on the SABiLSTM model, three commonly used IOAs, particle swarm optimization algorithm (PSO), crisscross optimization algorithm (CSO), and sparrow search algorithm (SSA), were employed to search for the optimal initialization parameters. The maximum iteration and population size of the three IOAs were selected as 50 and 100, respectively.

In addition, for health test dataset B, the quantitative evaluation metrics of the predicted results of three intelligent optimization algorithm models (IOAMs), SSA-SABiLSTM, PSO-SABiLSTM, and CSO-SABiLSTM, are presented in Table 6 and Table 7, as well as the ranked result (denoted as F_R_) of the Friedman test for the determination coefficient R^2^ based on different wind turbine datasets.

As can be observed in Table 6 and Table 7, by implementing intelligent optimizations in the SABiLSTM model using IOAs, the model’s performance presented a significant improvement. The average MAE, RMSE, and MAPE values of IOAMs were 30.78%, 27.23%, and 37.40% lower than those of the SABiLSTM network, respectively, while the R^2^ value of 3.07% was higher than that of the SABiLSTM network. Meanwhile, it can be observed in Table 6 and Table 7 that, among the three IOAs, the SSA presented the best performance improvement on the SABiLSTM network in terms of four mean metrics. The MAE, RMSE, and MAPE values of the SSA-SABiLSTM networks were 0.2543 °C, 0.3412 °C, and 0.0069, which were 47.23%, 42.19%, and 53.38% lower than those of the SABiLSTM network, respectively. The R^2^ value of the SABiLSTM network was 0.9731, which was 4.6% higher than that of the SABiLSTM network. Additionally, the ranked result of the Friedman test for the determination coefficient R^2^ also illustrated the superiority of the proposed SSA-SABiLSTM model, which ranked first among the three comparative models.

**Figure 11:**
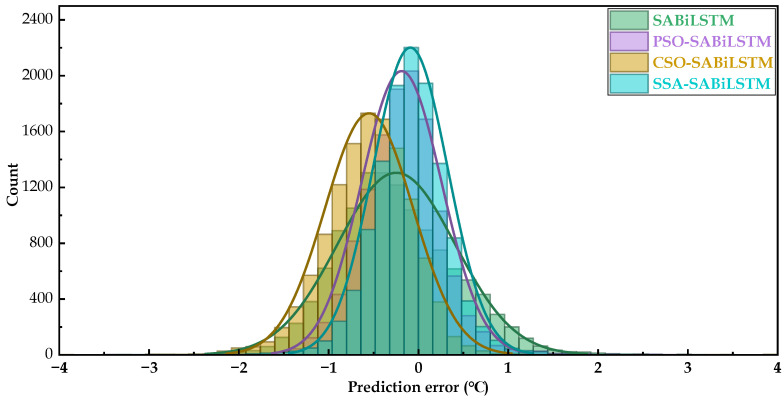
Probability density distribution of prediction errors of different NBMs for the main bearing temperature of WT No.20.

Figure 11 presents the probability density distribution of the predicted residuals of the SABiLSTM and IOAMs models (i.e., PSO-SABiLSTM, CSO-SABiLSTM, and SSA-SABiLSTM) for WT No.20. As can be observed in Figure 11, when compared to the SABiLSTM network, the PDF curves of IOAMs were sharper around the zero-error interval, presenting peaks, which indicated higher proportions of small residuals and more centralized residual distributions. Among the three PDF curves, the curve generated by the SSA-SABiLSTM network was the sharpest and most centralized compared to the other two, which validated the superiority of the SSA model in improving the model’s overall performance.

**Figure 12:**
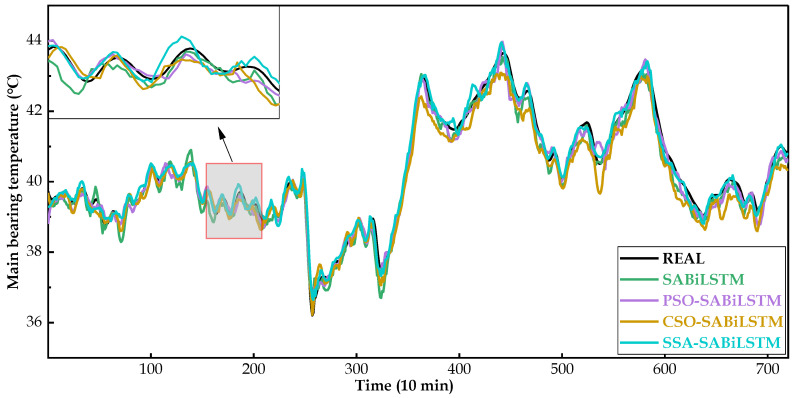
Main bearing predicted temperature values of different NBMs for WT No.20.

**Figure 13:**
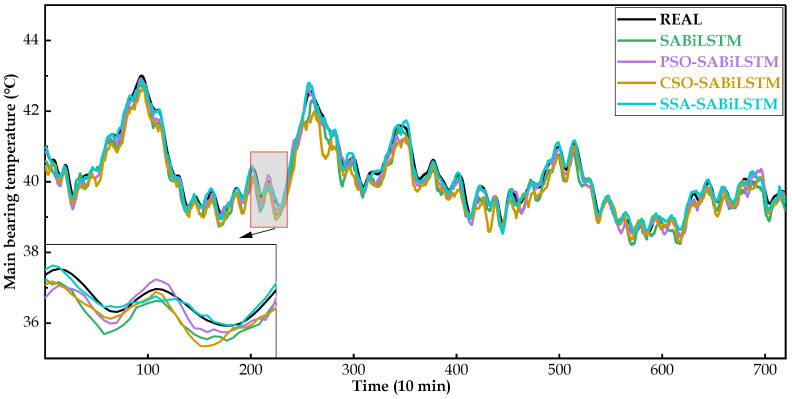
Main bearing predicted temperature values of different NBMs for WT No.23.

**Figure 14:**
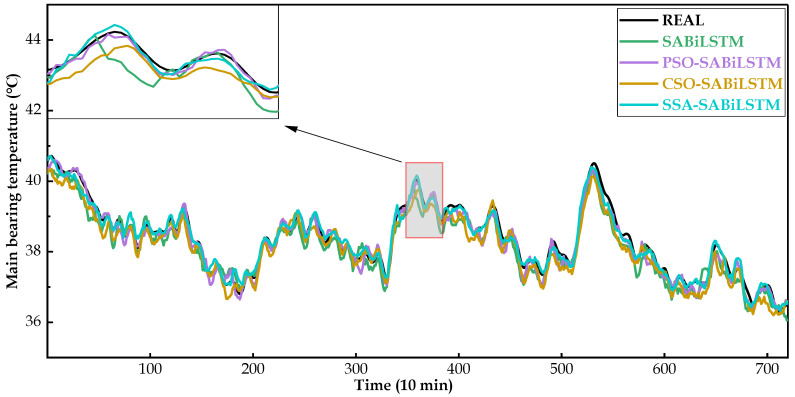
Main bearing predicted temperature values of different NBMs for WT No.26.

For the main bearing temperature of WTs No.20, No.23, and No.26, Figure 12, Figure 13 and Figure 14 intuitively present the real measured and predicted values generated by the SABiLSTM model and different IOAMs. It can be observed in Figure 12, Figure 13 and Figure 14 that, compared with the SABiLSTM model, the predicted values of IOAMs are closer to the real values, and the predicted curve of the SSA-SABiLSTM model is the smoothest, as well as the closest, to the real curve, which validates the effectiveness of different IOAs in the model performance improvement and the superiority of the SSA-SABiLSTM model among the three IOAMs.

**Figure 15:**
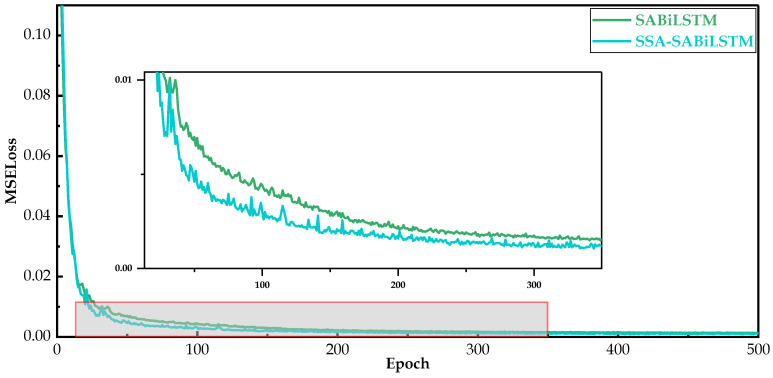
Training losses of SABiLSTM and SSA-SABiLSTM models.

As for the optimization effect of the model convergence speed, Figure 15 displays the training losses of the SABiLSTM and SSA-SABiLSTM models, from which we can observe the beneficial impact of the SSA model on the model training speed and model training difficulty. It can be observed in Figure 15 that the SSA-SABiLSTM model with a higher convergence speed converged after 300 iterations, while the SABiLSTM model showed a continuous declining trend in training loss values and did not converge, which verifies that the introduction of the SSA model can accelerate the convergence speed and reduce the training difficulty values.

In summary, based on the modeling datasets (i.e., A and B) collected from multiple wind turbines, the verification and comparative analysis conducted between the proposed SSA-SABiLSTM model and contrast models was performed in terms of the evaluation metrics, frequency distribution, and intelligent optimization algorithm. The analysis results illustrate that the constructed SSA-SABiLSTM model has a superior prediction performance and can better learn the normal behaviors of wind turbine main bearings when operating under normal conditions.

### 5.3. Wind Turbine Condition Monitoring

Based on the well-trained SSA-SABiLSTM model constructed and presented above, as well as the proposed anomaly detection method (i.e., BinSeg changepoint detection system and threshold alarm) described in Section 4, the main bearing condition monitoring could be performed to successfully identify deterioration conditions and detect potential faults in advance. In this sub-section, a real fault dataset (i.e., fault dataset **C**), which consisted of two main bearing failure cases acquired from WTs No.14 and No.27, was used to verify the superiority and effectiveness of the designed SSD approach. According to the operation and maintenance (O&M) records of the wind farm, the main bearings of WTs No.14 and No.27 experienced over-temperature faults and the SCADA system issued alarm signals at 6/9/2020 16:32 and 8/17/2020 11:21, respectively. 

**Figure 16:**
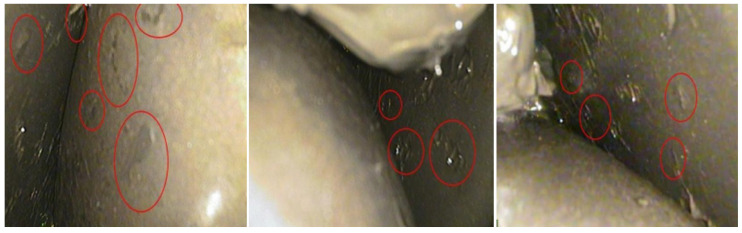
Damage of the roller, inner raceway, and outer raceway.

With further endoscopic explorations, it can be observed in Figure 16 that the roller, inner raceway, and outer raceway of the main bearings suffered from serious damage, which may be caused by excessive instantaneous loads due to extreme wind conditions.

#### 5.3.1. Identification of Deterioration Conditions

**Figure 17:**
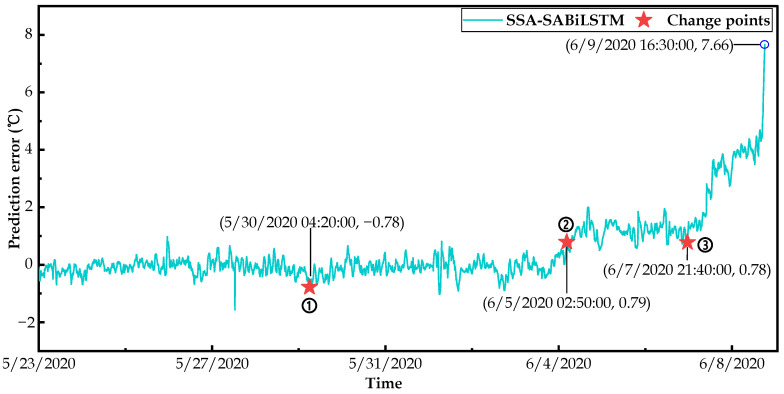
Result of changepoints detection for WT No.14.

**Figure 18:**
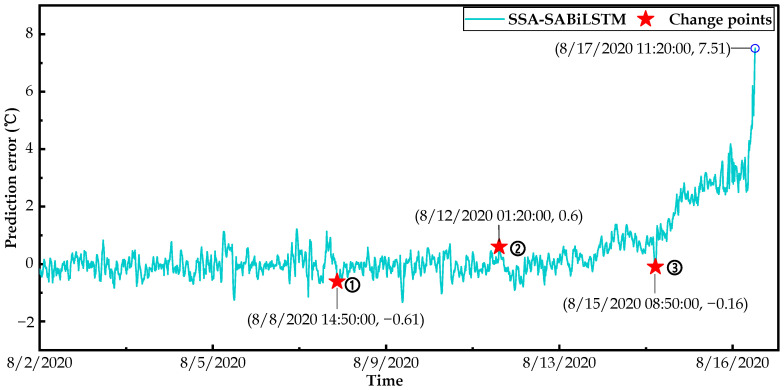
Result of changepoints detection for WT No.27.

Based on the well-trained SSA-SABiLSTM model and fault dataset C, the changepoint detection results of the predicted residuals of the main bearing temperature values for failure WTs No.14 and No.27 are respectively displayed in Figure 17 and Figure 18, where it can be observed that three changepoints are detected for two WTs. 

To ensure the accuracy and reliability of the identification of main bearing deterioration conditions, we took the last two changepoints into account in this study. As can be observed in Figure 17, the predicted residual value of WT No.14 was relatively low and slightly fluctuated at around 0 °C before 6/5/2020 02:50 (changepoint 2). Then, it gradually increased and fluctuated at around 1 °C between 6/5/2020 02:50 and 6/7/2020 21:40 (changepoint 3), and finally surged to 7.66 °C at 6/9/2020 16:30 when the SCADA system issued an alarm signal. As for WT No.27, the predicted residual displayed in Figure 18 was relatively small and slightly fluctuated at around 0 °C before 8/12/2020 01:20 (changepoint 2). Then, it gradually increased between 8/12/2020 01:20 and 8/15/2020 08:50 (changepoint 3), and finally surged to 7.51 °C at 8/17/2020 11:20 when the SCADA system issued an alarm signal.

In summary, from the residual sequence changepoint detection results obtained for WTs No.14 and No.27, it may be concluded that the main bearings deteriorated prior to the SCADA system issuing alarm signals. In detail, compared with the failure time recorded in the SCADA system, changepoints 2 and 3 detected in the residual sequence could identify the main bearing deterioration conditions 109.7 h and 42.87 h in advance for WT No.14 and 130.02 h and 50.52 h for WT No.27.

#### 5.3.2. Early Fault Warning

In this study, we not only implemented changepoint detection methods in the residual sequence, but we also conducted a statistical analysis according to the kernel density estimation (KDE) algorithm described in Section 4.2. Specifically, we first calculated the probability density function (PDF) of the predicted residual for wind turbines operating under normal conditions; then, we determined the alarm threshold of wind turbine condition monitoring to detect potential faults in advance. Then, based on the well-trained SSA-SABiLSTM model and health dataset B, the alarm threshold could be calculated as 2.13 °C according to Equations (31) and (32).

**Figure 19:**
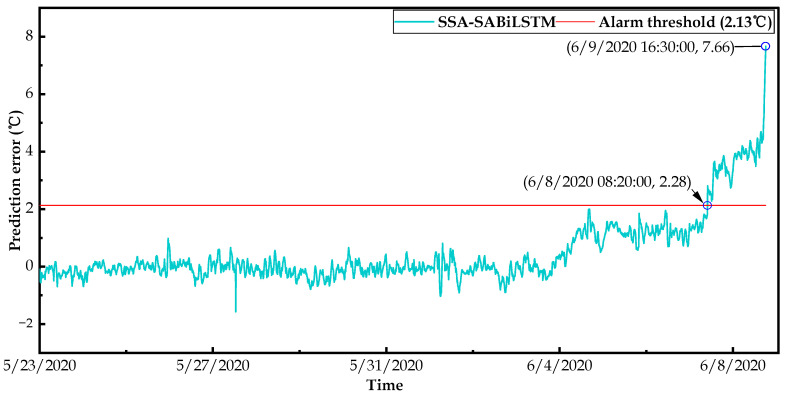
Results of changepoints detection and early fault warning for WT No.14.

**Figure 20:**
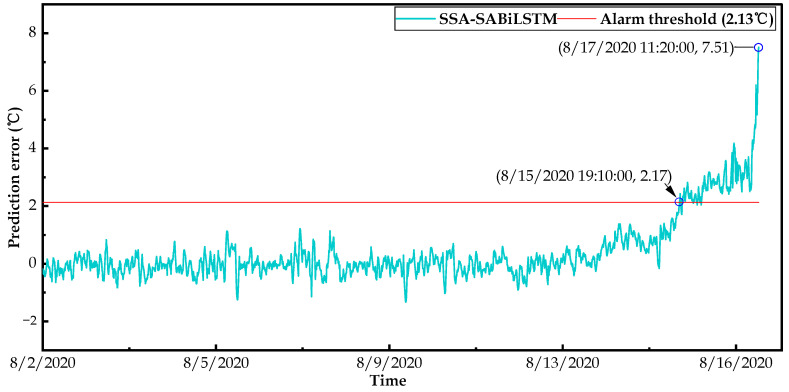
Results of changepoints detection and early fault warning for WT No.27.

Figure 19 and Figure 20 display the anomaly detection results of failure WTs No.14 and No.27. It can be observed in Figure 19 and Figure 20 that the predicted residuals of two WTs exceeded the alarm threshold at 6/8/2020 08:20 and 8/15/2020 19:10, respectively. Then, the residuals urged to the maximum values at 6/9/2020 16:30 and 8/17/2020 11:20, respectively, corresponding to the alarm time (i.e., 6/9/2020 16:32 and 8/17/2020 11:21) recorded in the SCADA system.

Consequently, compared with the actual failure times of WTs No.14 and No.27, the alarm threshold calculated in this study detected the potential main bearing over-temperature faults approximately 32.2 h and 40.18 h in advance, which verified the rationality of the determined alarm threshold and the early fault-detection capability of the designed SSD approach.

To sum up, based on fault dataset C collected from two wind turbines, the effectiveness and practicability of the designed SSD approach was validated in terms of the identification of the main bearing deterioration conditions and their early fault warnings. Moreover, the validation results illustrate that the designed SSD WTCM approach can automatically detect the deterioration conditions 47–120 h in advance and the potential faults approximately 36 h ahead of the occurrence of an actual fault, which can provide sufficient time for the O&M technicians to take appropriate measures (e.g., timely repairing or replacing) to avoid possible major accidents, unnecessary O&M costs, and abundant downtime results.

## 6. Conclusions

In this study, a novel wind turbine condition monitoring method (SSA-SABiLSTM-BinSegCPD, SSD) was proposed to automatically identify deterioration conditions and provide an early fault warning. Based on the datasets collected from multiple WTs, the effectiveness and superiority of the designed SSD approach was verified by comparing it with other models. The following conclusions can be drawn from the above experimental results.

(1) A normal-behavior model (SSA-SABiLSTM) for wind turbine critical components or subsystems was constructed by combining the sparrow search algorithm (SSA) and BiLSTM network with the self-attention mechanism (SA). The SSA-SABiLSTM model can effectively learn the nonlinear temporal dynamics characteristics hidden in the SCADA data. The introduction of the SA and SSA methods significantly improved the predicted performance of the BiLSTM model. The MAE, RMSE, and MAPE values of the SSA-SABiLSTM model are 0.2543 °C, 0.3412 °C, and 0.0069, which were 49.77%, 48.56%, and 54.1% lower than those of the BiLSTM model, respectively. The R^2^ value of the SABiLSTM model was 0.9731, which was 7.51% higher than that of the SABiLSTM model.
(2) A hybrid anomaly detection strategy consisting of the changepoints detection and threshold alarm was designed, which can improve the accuracy, reliability, and timeliness of the early fault warnings.
(3) A real fault dataset (i.e., fault dataset C) consisting of two actual main bearing failure cases was employed to verify the effectiveness and practicability of the SSA-SABiLSTM model and the hybrid strategy. The results illustrate that, compared with the failure time recorded by the SCADA system, the proposed SSD method can automatically identify the deterioration conditions 47–120 h in advance and detect the potential faults approximately 36 h ahead of the occurrence of the actual fault.

Additionally, except for main bearings, the proposed SSD method can be used for other key components (e.g., generator, gearbox) or even other machinery different to wind turbines. It also should be noted that this study only considered the SCADA operating data with a 10 min sampling interval without considering other monitoring signals, such as vibration signal. Therefore, in future studies, we aim to focus on the feature integration of 10 min SCADA and vibration signal data, taking these two types of data as model input parameters to improve the overall model’s performance.

## Figures and Tables

**Table 1:** Modeling datasets.

Datasets	Wind Turbines	Start Time (Month/Date/Year)	End Time (Month/Date/Year)	Fault Time (Month/Date/Year)	Raw Data	Valid Data
Health training dataset A	No.11	1/1/2020 0:00	1/1/2021 0:00	——	153,162	126,189
	No.15					
	No.19					
Health test dataset B	No.20	1/1/2020 0:00	4/20/2020 9:00	——	15,895	12,411
	No.23	5/1/2020 0:00	9/20/2020 8:40	——	20,501	12,304
	No.26	8/1/2020 0:00	11/18/2020 20:10	——	15,819	12,540
Fault dataset C	No.14	5/9/2020 0:00	6/9/2020 16:30	6/9/2020 16:32	4564	3657
	No.27	7/17/2020 0:00	8/17/2020 11:20	8/17/2020 11:21	4533	3781

**Table 2:** Result of variable selection.

No	Variables	Units	|R_P_|	No	Variables	Units	|R_P_|
1	Hub temperature	°C	0.7450	9	Active power	kW	0.3846
2	Ambient temperature	°C	0.7001	10	Gearbox rear bearing temperature	°C	0.3649
3	Control cabinet temperature	°C	0.5542	11	Wind speed	m/s	0.3504
4	Gearbox inlet oil temperature	°C	0.4869	12	Main shaft speed	rpm	0.3439
5	Gearbox oil temperature	°C	0.4612	13	Blade 3 motor temperature	°C	0.3409
6	Nacelle temperature	°C	0.4454	14	Gearbox inlet oil pressure	bar	0.3395
7	Gearbox front bearing temperature	°C	0.4220	15	Generator front bearing temperature	°C	0.3392
8	Generator rear bearing temperature	°C	0.3924	16	Generator stator winding temperature phase W	°C	0.3212

**Table 3:** Hyper-parameters of RNNs.

Hyper-Parameters	Algorithms/Values	Hyper-Parameters	Algorithms/Values
Loss function	MSE	Number of steps	8
Optimization algorithm	Adam	Number of epochs	1000
Batch size	64	Leaning rate	0.001

**Table 4:** Evaluation metrics of different NBMs for different wind turbines.

Model	Metrics											
	No.20				No.23				No.26			
	R^2^	MAE	RMSE	MAPE	R^2^	MAE	RMSE	MAPE	R^2^	MAE	RMSE	MAPE
XGBoost	0.8044	0.7011	0.9111	0.0184	0.8122	0.6870	0.8929	0.0180	0.7965	0.7151	0.9294	0.0187
BPNN	0.8345	0.6450	0.8382	0.0169	0.8416	0.6310	0.8200	0.0165	0.8485	0.6169	0.8018	0.0162
LSTM	0.8804	0.5389	0.7061	0.0114	0.8653	0.5819	0.7562	0.0152	0.8748	0.5608	0.7289	0.0147
BiLSTM	0.9093	0.4867	0.6378	0.0182	0.8883	0.5256	0.6885	0.0137	0.8963	0.5066	0.6636	0.0132
SABiLSTM	0.9314	0.4681	0.5687	0.0191	0.9218	0.4978	0.6120	0.0128	0.9280	0.4798	0.5899	0.0124

**Table 5:** Evaluation metrics of different NBMs.

Model	Mean Metrics for No.20, No.23, and No.26			
	R^2^	MAE	RMSE	MAPE
XGBoost	0.8044	0.7011	0.9111	0.0184
BPNN	0.8415	0.6310	0.8200	0.0165
LSTM	0.8735	0.5605	0.7304	0.0158
BiLSTM	0.8980	0.5063	0.6633	0.0150
SABiLSTM	0.9271	0.4819	0.5902	0.0148

**Table 6:** Evaluation metrics of different IOAMs for different wind turbines.

Model	Metrics														
	No.20					No.23					No.26				
	R^2^	F_R_	MAE	RMSE	MAPE	R^2^	F_R_	MAE	RMSE	MAPE	R^2^	F_R_	MAE	RMSE	MAPE
PSO-SABiLSTM	0.9637	2	0.2816	0.3723	0.0083	0.9619	2	0.303	0.4023	0.008	0.9646	2	0.292	0.3877	0.0079
CSO-SABiLSTM	0.9441	3	0.4346	0.5369	0.0141	0.9303	3	0.4726	0.5817	0.0123	0.9359	3	0.4555	0.5607	0.0119
SSA-SABiLSTM	0.9764	1	0.2473	0.3278	0.0072	0.9704	1	0.2626	0.3543	0.0069	0.9725	1	0.2531	0.3415	0.0066

**Table 7:** Evaluation metrics of different IOAMs.

Model	Mean Metrics of No.20, No.23, and No.26				
	R^2^	F_R_	MAE	RMSE	MAPE
PSO-SABiLSTM	0.9634	2	0.2922	0.3874	0.0081
CSO-SABiLSTM	0.9368	3	0.4542	0.5598	0.0128
SSA-SABiLSTM	0.9731	1	0.2543	0.3412	0.0069

## Data Availability

Not applicable.
